# Refractive Outcomes of Simultaneous Pterygium and Cataract Surgery With Fibrin Glue

**DOI:** 10.7759/cureus.19857

**Published:** 2021-11-24

**Authors:** Bharti Sharma, Sushil Kumar Bajoria, Minakshi Mishra, Nazmin Iqubal

**Affiliations:** 1 Ophthalmology, Tata Main Hospital, Jamshedpur, IND; 2 Pathology, Tata Main Hospital, Jamshedpur, IND

**Keywords:** intraocular lens, refraction, astigmatism, fibrin glue, cataract, pterygium

## Abstract

Introduction

Pterygium is commonly reported in India because of its tropical location. It is often accompanied by senile cataract. The current study aimed to evaluate the refractive outcomes of patients undergoing simultaneous pterygium and cataract surgery.

Materials and methods

A total of 12 patients with cataract and pterygium underwent simultaneous phacoemulsification with a foldable intraocular lens (IOL) followed by pterygium excision with conjunctival limbal autograft secured with fibrin glue under peribulbar anesthesia. Nasal pterygium was present in all the cases, and its size ranged from 2.5 to 3.5 mm from the limbus. All the patients exhibited nuclear sclerotic cataract of grades 2 and 3.

Results

The results of the patients were analyzed retrospectively. We determined the best-corrected visual acuity ([BCVA] in logMAR), manifest astigmatism, and mean corneal astigmatism before operation and after 12 months of surgery. We also analyzed the correlation between the prediction error (calculated by subtracting the predicted postoperative refraction from the actual postoperative spherical equivalent) and changes in the mean keratometry and prediction errors and pterygium size. The mean age of the patients was 61.9 ± 7.14 years (range: 50-70 years). The mean BCVA at presentation was 0.67 ± 0.24 logMAR units, which significantly increased after surgery to 0.01 ± 0.03 logMAR units (*p* < 0.001, Wilcoxon signed-rank test). The mean manifest corneal astigmatism significantly reduced from −1.98 ± 0.84 diopters (D) preoperatively to −0.54 ± 0.18 D postoperatively (*p* < 0.001). Mean corneal keratometry increased from 43.81 ± 1.77 D preoperatively to 44.19 ± 1.76 D postoperatively (*p* < 0.05). At 12 months, 58.33% and 41.66% of the eyes were within  ±0.5 D and  ±1.0 D, respectively. A significant correlation was observed between the prediction errors and changes in the mean keratometry (Spearman signed-rank test, r = −0.65, *p *< 0.05) and pterygium size (Spearman correlation coefficient, r = −0.75, *p* < 0.05).

Conclusion

The simultaneous pterygium and cataract surgery with conjunctival autograft and fibrin glue was safe and effective in providing excellent and predictable refractive outcomes. Steepening of the cornea after combined surgery results in myopia; therefore, the IOL power should be selected appropriately. The use of fibrin glue in surgeries reduces the operative time and postoperative pain and results in early postoperative rehabilitation of patients.

## Introduction

Pterygium is a triangular or wing-shaped fibrovascular growth of the conjunctiva, which encroaches onto the cornea and affects one or both eyes. It is a potentially blinding disease in the advanced stage due to invasion of the visual axis, which can have a significant impact on vision, and may require surgery for visual rehabilitation [1,2]. Pterygium excision with conjunctival autograft is the definitive treatment for pterygium and is associated with a low risk of recurrence [3]. Fibrin glue is used across several centers worldwide for adhesion of conjunctival autograft in pterygium surgery. Fibrin glue is a biological tissue adhesive, which imitates the final stages of the coagulation cascade when a solution of human fibrinogen is activated by thrombin (the two components of fibrin glue) and converted to fibrin, resulting in its adhesive capability. Thrombin also activates factor XIII (present in the fibrinogen component of the glue). Factor XIII cross-links and stabilizes the clot's fibrin monomers, while aprotinin inhibits fibrinolytic enzymes, consequently resulting in a stable clot. Fibrin glue reduces the operating time, postoperative pain, and discomfort, and results in early postoperative rehabilitation of patients compared with sutures [4]. Pterygium commonly occurs in older patients and is usually accompanied by cataract [5,6]. Removal of pterygium can be performed either before or subsequently with cataract surgery. Sequential pterygium excision followed by cataract surgery has been the preferred strategy. This approach has advantages over combined pterygium and cataract surgery in terms of better corneal stability and subsequent higher predictability of intraocular lens (IOL) power calculation [7]. However, most of the patients prefer single-step combined procedures rather than two-step separate procedures. Simultaneous cataract and pterygium surgery provides faster visual recovery and cosmetic improvement and reduces hospital visits and the overall treatment cost. [8-10]. The current study aimed to assess the refractive outcomes of simultaneous cataract and pterygium surgeries using fibrin glue.

## Materials and methods

We retrospectively reviewed the results of 12 consecutive patients who had undergone simultaneous phacoemulsification with foldable IOL implantation and pterygium excision with conjunctival limbal autograft using fibrin glue. Patients with a history of ocular trauma and surgeries, glaucoma, retinal abnormalities, double-headed pterygiums, and those that interfered with keratometry reading were excluded from the study. Written informed consent was obtained from all the patients for surgery after explaining to them the nature and possible consequences of the study. All the patients underwent complete ophthalmic examination. Slit lamp and dilated fundus examination was performed in all the patients. Best-corrected visual acuity (BCVA) and manifest refraction, including astigmatism and keratometry values, were recorded preoperatively and 12 months after surgery. The preoperative horizontal size of the pterygium from the limbus to the advancing head or pterygium apex was measured in millimeters by focusing the horizontal slit beam and using a ruler incorporated in the slit lamp microscope. Each measurement was repeated at least three times, and the mean value was used for the analysis. Only patients with a year of follow-up were included in the study. Keratometry values of vertical and horizontal meridian were recorded using an automated keratometer (KR 800, Topcon Healthcare, Tokyo, Japan). IOL power was calculated using the SRK/T formula, and the axial length was measured through immersion biometry (OcuScan® RxP, Alcon, Fort Worth, TX). The Wilcoxon signed-rank test was used to compare the pre- and postsurgical data. The results are expressed as mean ± SD, and a p-value of <0.05 was considered statistically significant. For all the patients, we selected emmetropia as the target refraction. The prediction errors were calculated by subtracting the predicted postoperative refraction from the actual postoperative spherical equivalent 12 months after surgery. The relationship between prediction errors and changes in mean keratometry and pterygium size was analyzed using Spearman’s rank correlation test.

Surgical technique

All surgeries were performed under peribulbar anesthesia by a single surgeon. All the patients first underwent standard phacoemulsification (capsulorhexis, nucleus and cortex extraction) with a monofocal IOL (AcrySof® IQ, Alcon Laboratories Ireland Ltd., Cork, Ireland) implantation. The surgery was performed by making a 2.8-mm clear corneal, superonasal incision in the left eye and a superotemporal incision in the right eye. Subconjunctival dissection of the pterygium was then performed, starting dissection at the neck of the pterygium. The pterygium head was dissected from the cornea by using a crescent blade, and the body was excised and sent for histopathological examination. Minimal cautery was applied to the bleeding points. The bare sclera area was measured. The corresponding size of the conjunctival autograft was taken from the superior bulbar conjunctiva and adhered over bare sclera with fibrin glue (TISSEEL Lyo, Fibrin Sealant kit, Baxter Healthcare SA, Zurich, Switzerland). Postoperatively, the patients were advised to patch between topicals until the corneal epithelial defect healed. All the patients were prescribed topical prednisolone acetate 1% eye drops, six times a day, in tapering doses for one month, moxifloxacin 0.5% eye drops, four times a day for two weeks, and hydroxypropyl methylcellulose 0.3% eye drops four times a day for one month.

## Results

All the patients had nasal pterygium extending from 2.5 to 3.5 mm on the cornea from the limbus and nuclear sclerosis of grade 2/3 with a posterior subcapsular cataract of variable densities. The mean age of the patients was 61.9 ± 7.14 years (range: 50-70 years). Overall, nine (75%) men and three (25%) women were included in the study (Table 1).

**Table 1:** Preoperative demographics of patients undergoing simultaneous pterygium and cataract surgery D, diopter; LogMAR, logarithm of the minimal angle of resolution; BCVA, best-corrected distant visual acuity

		Preoperative demographics (mean standard deviation)
	Male:female	9:3
	Age	61.9 ± 7.14 years (range: 50-70 years)
	Astigmatism	–1.979 ± 0.84 D
	Mean keratometric reading	43.81 ± 1.77 D (range: 40.88-45.75 D)
	LogMAR BCVA	0.67 ± 0.24 (range: 0.3-1)

**Figure 1:**
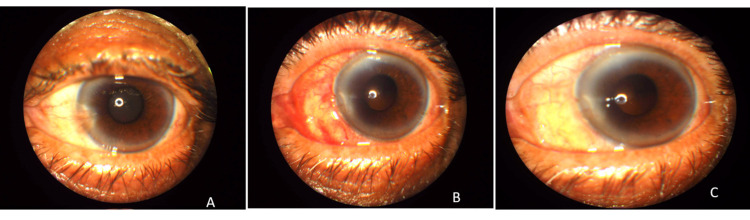
(A) Patient with pterygium and cataract preoperatively. (B) Postoperative day 1 after simultaneous cataract and pterygium surgery with fibrin glue. (C) Postoperative 12 months after simultaneous cataract and pterygium surgery with fibrin glue.

All surgeries were uneventful, and no intraoperative or postoperative complication was observed (Figure 1).

**Figure 2:**
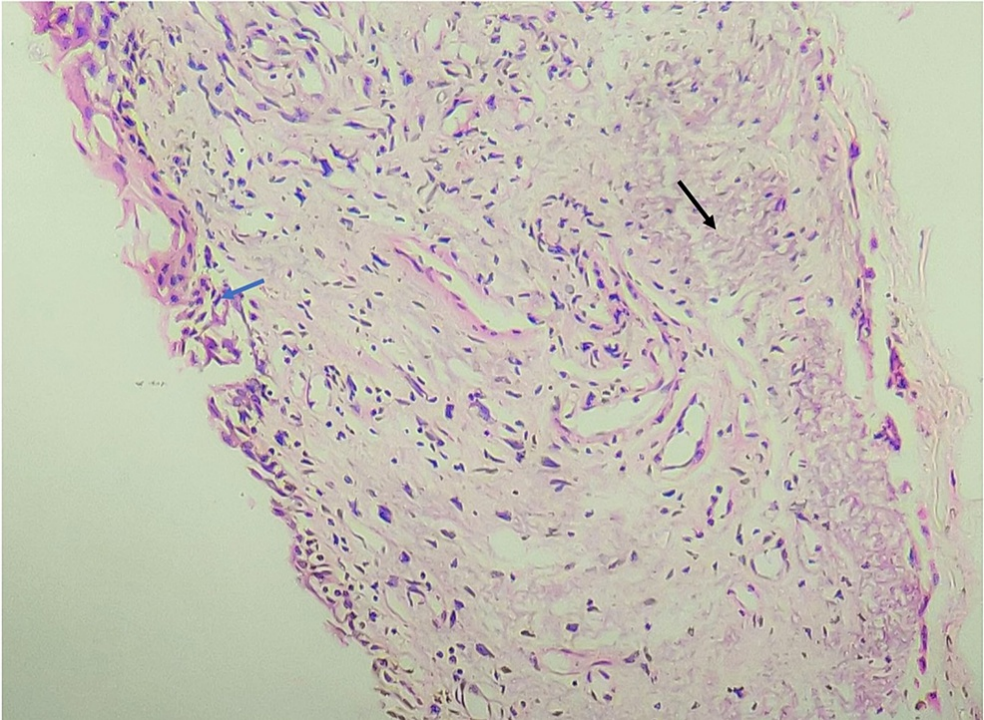
H&E (x200). Microphotograph showing preserved squamous epithelium with intact basement membrane on the left (blue arrow), well-vascularized stroma, and sparsely cellular basophilic fibrillary degeneration of the fibroelastic tissue (black arrow). H&E, hematoxylin and eosin

Excised pterygium tissue revealed preserved squamous epithelium with intact basement membrane, well-vascularized stroma, and sparsely cellular basophilic fibrillary degeneration of the fibroelastic tissue (Figure 2).

**Figure 3:**
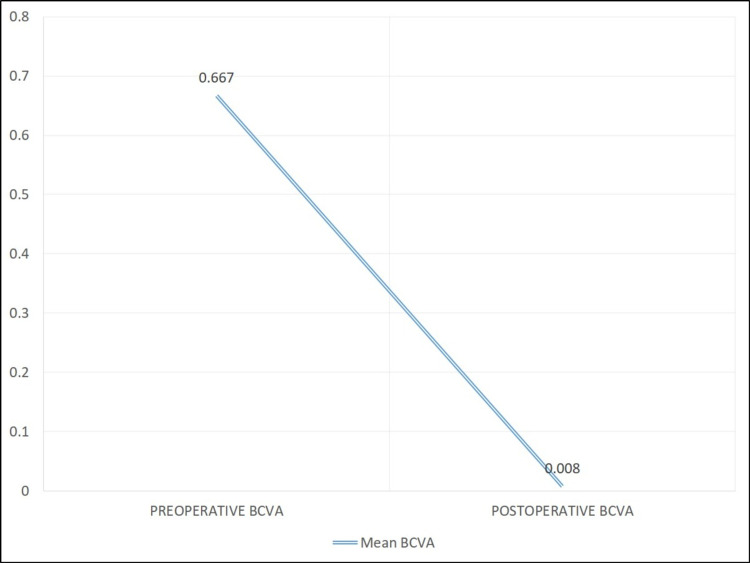
Changes in mean BCVA after simultaneous pterygium and cataract surgery. BCVA, best-corrected visual acuity

The mean BCVA significantly improved from 0.667 ± 0.24 logMAR unit (range: 0.3-1 logMAR unit) preoperatively to 0.008 ± 0.03 logMAR unit postoperatively after one year (p < 0.01) (Figure 3).

**Figure 4:**
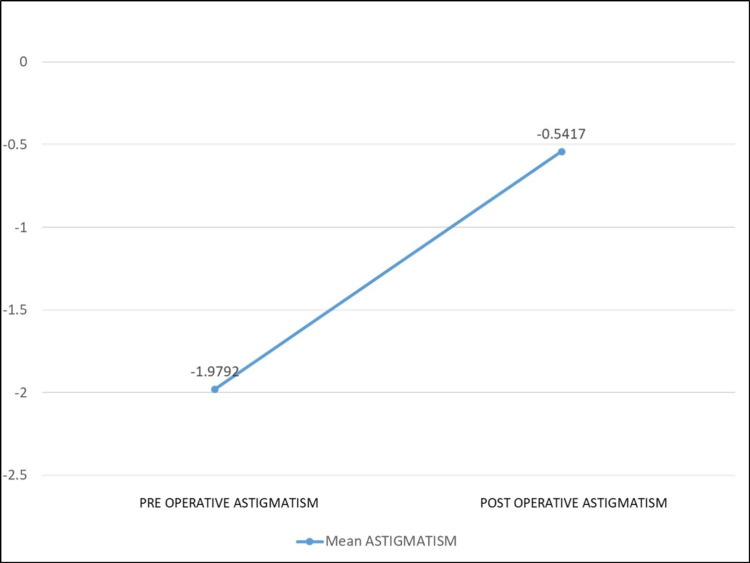
Changes in mean corneal astigmatism after simultaneous pterygium and cataract surgery.

The mean corneal astigmatism significantly decreased from −1.979 ± 0.84 D preoperatively to −0.54 ± 0.18 D one year after surgery (p < 0.01) (Figure 4).

**Figure 5:**
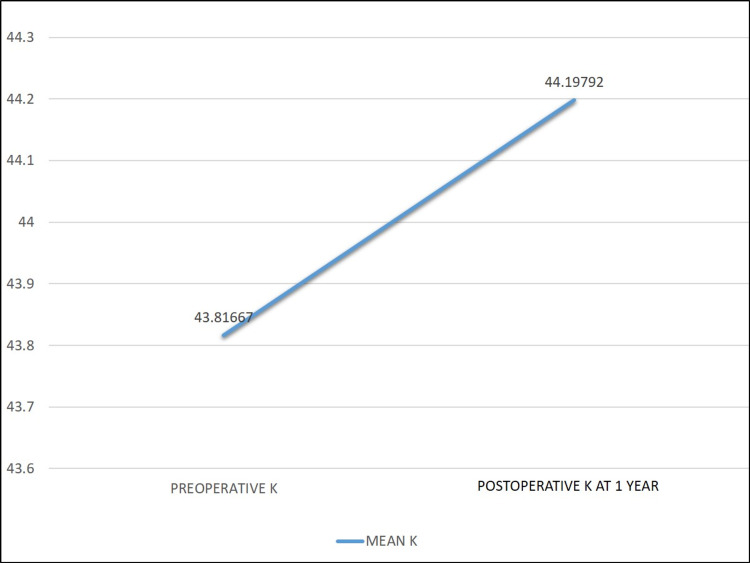
Changes in mean corneal keratometry after simultaneous pterygium and cataract surgery. K, keratometry

The mean keratometry reading increased from 43.81 ± 1.77 D preoperatively to 44.19 ± 1.76D one year after surgery (p < 0.05) (Figure 5).

A significant correlation was observed between the prediction errors and the changes in the mean keratometry values (Spearman correlation coefficient, r = −0.65, p < 0.05) and prediction errors and pterygium size (Spearman correlation coefficient, r = −0.75, p < 0.05). At 12 months, 58.33% and 41.66% of the operated eyes were within  ±0.5 and  ±1.0 D, respectively, of the targeted correction. The final achieved refraction (−0.54 ± 0.29 D) was significantly more myopic than the targeted refraction (0.00 D) (p < 0.01).

## Discussion

Exposure to UV light causes an increased incidence of cataract in patients with pterygium [5,11,12]. Cataract remains the leading cause of avoidable blindness worldwide [13]. A high incidence of pterygium due to exposure to UV light has been reported in the Indian subcontinent [14,15]. Definite treatment of pterygium includes its excision, and pterygium excision with conjunctival autograft remains the gold standard treatment with less incidence of recurrence. Pterygium excision can be performed before or in combination with cataract surgery. During sequential surgery, it is crucial to wait for stabilization of the cornea, which could take up to 4-12 months. Many patients prefer single-step combined pterygium excision with cataract surgery as it provides rapid visual recovery, reduces hospital visits, and is economical.

Our results revealed that combined cataract and pterygium surgery was safe and effective and provided reasonably predictable refractive results. In our study, significant improvement in BCVA was observed one year after surgery (p < 0.01). Refractive results of simultaneous pterygium and cataract surgery have been described in a limited number of studies. Ibechukwu [8] first described that simultaneous cataract and pterygium surgery was beneficial in terms of cost reduction and visual prognosis to both the hospital and patients; however, the refractive outcomes of these procedures were not described. Gulani and Dastur [9] stated that simultaneous cataract and pterygium surgery is beneficial for patients. Out of 30 patients undergoing simultaneous surgery, 63% patients exhibited visual recovery to 6/12, and the mean with-the-rule and against-the-rule astigmatism, was 1.3 D and 1.2 D, respectively, six months postoperatively. However, intracapsular/extracapsular cataract extraction was performed with suture application. Kamiya et al. [10] assessed the predictability of IOL power calculation after simultaneous pterygium excision and cataract surgery three months after surgery. They performed phacoemulsification and used 8-0 absorbable polyglycolic sutures to secure the conjunctival graft and reported significant improvement in BCVA, decreased manifest astigmatism, and increased mean keratometry after three months (p < 0.01). The percentages of eyes within ±0.5 D and ±1.0 D of targeted correction at three months were 48% and 82%, respectively. In our study, the percentage of patients within ±0.5 D of the targeted correction was slightly higher than in the study by Kamiya et al. This difference could probably be due to the progressive reduction in HOAs (higher-order aberrations) after pterygium surgery. Moreover, the final analysis in our study was performed 12 months after surgery, whereas Kamiya et al. performed the analysis three months after surgery. Gumus et al. revealed that the root mean square values of total aberrations and total HOAs were significantly decreased at three and 12 months after uneventful pterygium surgery with conjunctival autograft [16].

We also observed a moderate but significant increase in the mean keratometry postoperatively (p < 0.05). We further demonstrated that the increase in keratometry was significantly associated with the postoperative prediction errors. These findings indicate that the postoperative myopic shift occurred due to steepening of the cornea after pterygium removal, as pterygium tends to flatten the cornea. Our results are consistent with those of previous studies on changes in the corneal power after pterygium excision [16-18].

Additionally, our subgroup analysis revealed a significant correlation between the pterygium size and the prediction errors, which suggested that a sizeable myopic shift occurs when the pterygium is large. Tomidokoro et al. reported that pterygium removal with a 20% extension on the cornea will lead to a 0.91 D increase in corneal spherical power. Still, the prediction may contain considerable inaccuracy [17]. Kim et al. illustrated a significant correlation between the length of pterygium and the change in the mean keratometry after pterygium removal, although the pterygium of length < 2.0 mm rarely induced postoperative changes in the cornea [18]. Nejima et al. also reported that topographic changes were associated with pterygium size after pterygium excision in a study of 562 patients [19].

We used fibrin glue in all our cases to reduce surgery time and to enhance patient comfort. Wadgaonkar et al. revealed that fibrin glue in pterygium surgery significantly reduced operative time and provided considerable comfort to patients [4]. Various studies on pterygium excision have revealed that fibrin glue may result in less recurrence and may take less time than sutures to fix the conjunctival graft in place during pterygium surgery [20]. The current study has several limitations. First, the sample size of the study was small. Second, the corneal topographic changes and HOAs were not assessed in this study. Third, the outcomes of combined surgery were not compared with those of sequential pterygium and cataract surgeries. These surgeries depend on the skills of the surgeon. Therefore, comparing the postsurgical data of the two groups would be clinically helpful.

## Conclusions

Our study revealed that combined pterygium and cataract surgery with conjunctival autograft using fibrin glue is safe and provides predictable refractive outcomes. Although combined pterygium and cataract surgery significantly reduces refractive astigmatism, the postoperative myopic shift observed in these cases is due to the increase in steepness of the cornea, and this should be kept in mind preoperatively while selecting the IOL. Conjunctival graft adhesion with fibrin glue is an ideal alternative to suturing in these combined cases because it shortens the operative time, is easy to use, and is associated with less postoperative inflammation and discomfort. Simultaneous pterygium excision with cataract surgery offers specific advantages over sequential surgery and should be considered as a feasible option for elderly patients, especially when the size of pterygium is not large.
